# Alpha band oscillations in common synaptic input are explanatory of the complexity of isometric knee extensor muscle torque signals

**DOI:** 10.1113/EP092031

**Published:** 2024-08-20

**Authors:** Christopher R. J. Fennell, Alexis R. Mauger, James G. Hopker

**Affiliations:** ^1^ School of Sport and Exercise Sciences University of Kent Canterbury UK

**Keywords:** common synaptic input, muscle fatigue, muscle torque, physiological complexity

## Abstract

We investigated whether the strength of oscillations in common synaptic input was explanatory of knee extensor (KE) torque signal complexity during fresh and fatigued submaximal isometric contractions, in adults aged from 18 to 90 years. The discharge times of motor units were derived from the vastus lateralis muscle of 60 participants using high‐density surface EMG, during 20 s isometric KE contractions at 20% of maximal voluntary contraction, performed before and after a fatiguing repeated isometric KE contraction protocol at 60% of maximal voluntary contraction. Within‐muscle coherence Z‐scores were estimated using frequency‐domain coherence analysis, and muscle torque complexity was assessed using multiscale entropy analysis and detrended fluctuation analysis. Alpha band (5–15 Hz) coherence was found to predict 23.1% and 31.4% of the variance in the complexity index under 28‐scales (CI‐28) and detrended fluctuation analysis α complexity metrics, respectively, during the fresh contractions. Delta, alpha and low beta band coherence were significantly increased due to fatigue. Fatigue‐related changes in alpha coherence were significantly predictive of the fatigue‐related changes in CI‐28 and detrended fluctuation analysis α. The fatigue‐related increase in sample entropy from scales 11 to 28 of the multiscale entropy analysis curves was significantly predicted by the increase in the alpha band coherence. Age was not a contributory factor to the fatigue‐related changes in within‐muscle coherence and torque signal complexity. These findings indicate that the strength of alpha band oscillations in common synaptic input can explain, in part, isometric KE torque signal complexity and the fatigue‐related changes in torque signal complexity.

## Abstract (highlights)

- **What is the central question of this study?**
  Can the strength of oscillations in common synaptic input explain the complexity of isometric knee extensor torque signals?
- **What is the main finding and its importance?**
  Stronger alpha band oscillations in common synaptic input were associated with isometric knee extensor torque signals of higher complexity. Fatigue‐related changes in the torque signal complexity were associated with the fatigue‐related changes in the strength of alpha band oscillations in common synaptic input. These findings suggest that the complexity measures derived from isometric knee extensor torque signals reflect the neural mechanisms underpinning the modulation of torque control.

## 1 INTRODUCTION

Complex biological systems generate measurable non‐linear outputs that are neither completely random nor completely deterministic but exhibit both characteristics, falling along the spectrum from true randomness to perfect order. The torque or force output of an isometrically contracting human skeletal muscle when measured across time exhibits complex non‐linear fluctuations with temporal structure (Vaillancourt & Newell, 2002, 2003). The non‐linear characteristics of a muscle torque signal can be assessed using complexity metrics. These include the detrended fluctuation analysis (DFA), which measures the fractal scaling (i.e., statistical self‐similarity) of the signal, and the multiscale entropy analysis (MSE), which captures the time scale‐dependent structure of the torque signal (i.e., capturing the regularity or randomness of the torque signal across multiple time scales; Clark et al., 2023; Pethick et al., 2021a).

The complexity of a muscle torque signal is suggested to quantify the ability of the neuromuscular system to respond sufficiently to internal and external task demands, in addition to the adaptability of the system to stressors in an ever‐changing environment (Manor & Lipsitz, 2013; Peng et al., 2009). Accordingly, a well‐functioning neuromuscular system, able to meet or adapt to the imposed task demands, is expected to exhibit a torque output of high complexity and low variability. Conversely, a neuromuscular system producing a torque output of low complexity and high variability is suggested to be unable to respond effectively or adapt to task demands (Pethick et al., 2015, 2016, 2021b).

Motoneurons receive synaptic inputs (originating from afferent feedback, descending cortical and reticulospinal pathways, and neuromodulatory pathways from the brainstem; Enoka & Farina, 2021; Heckman & Enoka, 2012) that generate the effective neural drive to the muscle (Negro & Farina, 2011). Muscle torque is then produced through the filtering of the effective bandwidth of neural drive (<10 Hz) with the average twitch force of the active motor units (MUs). As independent synaptic inputs are filtered out by motoneurons, it is the low‐frequency common input components (the control input and common noise) received by the motoneurons that make muscle torque control possible (Farina & Negro, 2015). Moreover, research using cross‐correlation analyses has demonstrated that the low‐frequency oscillations (<10 Hz) in common synaptic input (i.e., effective neural drive) closely resemble the structure of the muscle torque signal (Dideriksen et al., 2012; Mazzo et al., 2022; Negro et al., 2009; Thompson et al., 2018).

Neuromuscular fatigue has been shown comprehensively to reduce the complexity of knee extensor (KE) torque during isometric contractions across multiple studies using a range of methodologies (for reviews, see Pethick et al., 2021b; Pethick & Tallent, 2022). In addition, age‐related reductions in neuromuscular complexity have also been observed across multiple muscle groups, including the KE muscles (Fiogbe et al., 2021; Knol et al., 2019; Pethick, 2023; Vaillancourt & Newell, 2003). Muscle torque complexity is noted to be of functional importance to the performance of fundamental motor skills and, consequently, active daily living tasks (Pethick et al., 2022). Despite the potentially deleterious impact of further reductions in muscle torque complexity induced by fatigue in older adults, the effect of ageing on fatigue‐related changes in KE complexity is yet to be established.

Maintenance of a constant muscle torque during fatiguing contractions requires neural strategies to compensate for decreases in the torque‐producing capacity of the muscle (Rossato et al., 2022). One such neural strategy is the increase in common synaptic input to the motoneurons innervating the fatigued muscles (Castronovo et al., 2015; Contessa et al., 2009; McManus et al., 2016; Rossato et al., 2022). The presence of common synaptic input is required for the effective regulation of muscle torque (Farina & Negro, 2015); however, too much common synaptic input and, consequently, MU synchronization, might be deleterious to motor control (Baker et al., 1992; Yao et al., 2000). Indeed, research has found that fatigue‐related increases in the low‐frequency bands of common synaptic input are explanatory of the increased variability of the fluctuations in isometric muscle torque [measured by the coefficient of variation of torque/force (CVT); Castronovo et al., 2015; Contessa et al., 2009; McManus et al., 2016]. Likewise, age‐related increases in the CVT have been associated with the increased strength of low‐frequency common synaptic input (Castronovo et al., 2018).

Based upon the evidence demonstrating that the low‐frequency oscillations in common synaptic input determine the variability of fluctuations in isometric torque signals (Castronovo et al., 2015; Farina et al., 2014; Negro et al., 2009), researchers have speculated that common synaptic inputs might be explanatory of the temporal structure of the muscle torque signal captured by the complexity metrics (Pethick et al., 2021b, 2016). Additionally, it has been postulated that the fatigue‐induced loss of muscle torque signal complexity might be the consequence of changes in common synaptic input (Pethick et al., 2021b, 2016). However, there has not yet been research to begin substantiating these hypotheses. Therefore, the new aims of the present study were to investigate: (1) whether the strength of oscillations in common synaptic input to the motoneuron pool could explain KE torque signal complexity during fresh submaximal isometric contractions; (2) whether the potential fatigue‐related changes in the strength of oscillations in common synaptic input could explain any fatigue‐related changes in KE torque signal complexity; and (3) whether age affects the strength of oscillations in common synaptic input to the motoneuron pool and therefore KE torque signal complexity during fresh and fatigued isometric contractions. The secondary aim of the study was to determine the strength of association between the effective neural drive to the vastus lateralis (VL) muscle and fluctuations in isometric KE torque during fresh and fatigued contractions.

We hypothesized that: (1) stronger oscillations in common synaptic input to the motoneuron pool would explain a lower KE torque signal complexity during fresh submaximal isometric contractions; (2) fatigue would increase the strength of oscillations in common synaptic input and would explain a fatigue‐related decrease in isometric KE torque control (i.e., lower complexity and higher variability torque signal); (3) older adults would exhibit stronger oscillations in common synaptic input to the motoneuron pool and therefore lower KE torque complexity during fresh and fatigued isometric contractions, in comparison to younger adults; and (4) effective neural drive would be strongly cross‐correlated with isometric KE torque during fresh and fatigued contractions.

## 2 METHODS

### 2.1 Ethical approval

Sixty healthy participants (56 male and 4 female) aged between 18 and 90 years were recruited to participate in the study. Participants were divided into three age groups: the younger age group (YG) were aged 18–30 years (*n *= 20; 17 male and 3 female; 23.9 ± 4.2 years); the middle age group (MG) were aged 31–49 years (*n *= 20; 19 male and 1 female; 40.9 ± 3.8 years); and the older age group (OG) were aged 50–90 years (*n *= 20; 20 male and 0 female; 63.3 ± 10.2 years).

All participants were regular exercisers, having performed above the World Health Organisation guidelines for ≥2 years (i.e., 2.5–5 h of moderate exercise per week; Bull et al., 2020). Participants were required to be non‐obese, non‐smokers, not to have previous or current circulatory disorders, to have no known signs or symptoms of cardiovascular, neuromuscular, renal or metabolic conditions and to be able to perform maximal exercise. The study was completed with full ethical approval of the University of Kent Research Ethics Committee (proposal number: 32_20_23), according to *Declaration of Helsinki* standards (but without being registered). All participants provided written informed consent and completed a health questionnaire prior to testing.

Participants were instructed to refrain from any exercise during the day prior to testing and from intense exercise in the 2 days prior. Participants were instructed to arrive euhydrated and in a post‐prandial state, having eaten ≥4 h prior to testing. Participants were told not to consume caffeine within 8 h and alcohol within 24 h of testing.

### 2.2 Experimental design

Each participant completed one experimental session consisting of the measurement of maximal voluntary isometric contraction (MVC) torque of the KE muscles, a task familiarization, and the measurement of fresh and fatigued KE muscle torque control, while high‐density surface electromyography (HD sEMG) signals were recorded from the VL muscle. Participant height and mass were also measured.

### 2.3 Measurement of the maximal voluntary isometric KE torque

Participants were seated on an isokinetic dynamometer (Cybex HUMAC Norm; CSMi, Stoughton, MA, USA), initialized and calibrated according to the manufacturer's instructions. The right leg of each participant was attached securely to the lever arm of the dynamometer, with the lateral epicondyle of the right femur in line with the axis of rotation of the lever arm. The knee angle was set at 90°, with full extension being 0°. Participants wore an over‐the‐shoulder and waist seat belt to prevent unwanted movement and use of hip extensors during the contractions. The computer monitor (60 cm in diameter) displaying the instantaneous torque was positioned 1 m in front of the eye line of the participants.

Once set up on the dynamometer, participants performed a warm‐up of 10 submaximal isometric contractions of increasing effort, after which a series of brief (6 s) MVCs were performed to establish maximum torque. The MVCs were repeated (separated by 60 s rest) until a plateau in peak torque was reached (i.e., until three consecutive peak torques were within 5% of each other). The highest torque value was recorded as the MVC, which was then used to set the isometric contraction intensities for the KE fatigue protocol. Participants then rested for 10 min before commencing the isometric KE fatigue protocol.

The KE torque data were recorded at 2048 Hz via a CED Micro 1401‐3 (Cambridge Electronic Design, Cambridge, UK), interfaced with a personal desktop computer, with data collected (Spike2, Cambridge Electronic Design) and exported at 1000 Hz for complexity analysis (Pethick et al., 2015) and at 2048 Hz for cross‐correlation analysis offline in MATLAB (R2023a; The MathWorks, Natick, MA, USA).

### 2.4 Isometric KE torque fatigue protocol

Prior to commencing the isometric KE fatigue protocol, participants were set up with the HD sEMG electrode grid on their right VL muscle. Participants were then familiarized with matching their instantaneous KE torque output to a thin line (1 mm thick) superimposed on the computer monitor at 10% and 20% of MVC. The *y*‐axis of the display was scaled to the MVC of each participant, ensuring that the superimposed line was located at the same position on the monitor for all participants.

**FIGURE 1:**
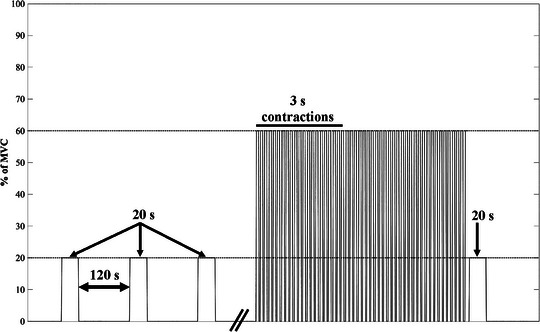
Schematic diagram of the isometric KE fatigue protocol. Participants completed three 20 s fresh isometric KE contractions at 20% of MVC with 120 s recovery, followed by repeated 3 s isometric KE contractions at 60% of MVC to task failure (or completion of 100 repetitions) with 2 s recovery between each contraction, immediately (2 s) followed by a ‘fatigued’ isometric KE contraction at 20% of MVC. Abbreviations: KE, knee extensor; MVC, maximal voluntary contraction.

After familiarization, participants performed three 20 s fresh isometric KE contractions at 20% of MVC, with 120 s recovery each. Contractions lasting 20 s were used to ensure that the target torque (20% of MVC) was met for >10 s, while ensuring that the contractions did not induce fatigue. The fresh isometric contractions were followed by repeated 3 s isometric KE contractions at 60% of MVC to task failure, with 2 s recovery between contractions. The isometric contraction intensity at 60% of MVC was selected to be above the critical torque for each participants (Pethick et al., 2016). Task failure was defined as the participant being unable to meet the required torque (60% of MVC) for two consecutive contractions. The task also ended if the participant completed 100 contractions. Eight of the 60 participants reached 100 contractions. Immediately (2 s) after task failure, the participant completed one 20 s isometric KE contraction at 20% of MVC, as the measure of fatigued KE torque (Figure 1). The time to task failure (TTF) of the 60% of MVC task was recorded as the measure of task performance.

The isometric KE contraction intensity was set at 20% of MVC for the measurement of fresh and fatigued KE torque control because this intensity demonstrated the highest yield of MUs from the VL muscle in pilot testing when the interference HD sEMG signals were decomposed.

### 2.5 High‐density sEMG

During all isometric KE contractions at 20% of MVC, HD sEMG signals were recorded from the VL muscle of the right leg using semi‐disposable 64‐electrode grids (5 rows × 13 columns; 4 mm electrode diameter; 8 mm interelectrode distance; GR08MM1305; OT Bioelettronica, Torino, Italy). Prior to the placement of the electrode grid, the skin of the VL muscle was clean shaven, firmly rubbed with medical grade abrasive paste (Spes Medica, Battipaglia, Italy), and cleansed with a 70% ethanol wipe.

The electrode grids were attached to the skin using disposable adhesive foam interfaces (F0A08MM1305; OT Bioelettronica), and the holes of the foam interfaces were filled with conductive paste to ensure skin–electrode contact (Spes Medica, Battipaglia, Italy). The electrode grids were secured further with kinesiology tape (Kinesio Precut, Albuquerque, NM, USA). The electrode grid was aligned longitudinally with orientation of the muscle fibres (proximal to distal) on the centre of the right VL muscle belly, which was determined via palpation. Skinfold thickness at the site of application of the electrode grid was determined before attachment using Harpenden skinfold callipers (British Indicators, Burgess Hill, UK).

The reference electrode was placed on the patella of the right leg and connected to the HD sEMG pre‐amplifier. Six earth electrodes (Ag/AgCl, 37.5 mm × 37.5 mm; Ambu WhiteSensor 4831Q) were attached to the participant and isokinetic dynamometer (Cybex HUMAC Norm; CSMi) to reduce baseline HD sEMG signal noise (Martinez‐Valdes et al., 2016). Three earth electrodes were attached to the participant: on the patella of the right leg, the styloid process of the ulna on the right arm and the malleolus of the right leg. Three further earth electrodes were attached directly around the base of the dynamometer chair.

The HD sEMG signals were recorded in monopolar mode, sampled at 2048 Hz, and converted to digital data by a 12‐bit analog‐to‐digital converter (EMG‐USB2+, 64‐channel EMG amplifier; OT Bioelettronica; 3 dB), bandpass filtered (10–500 Hz) and recorded in OTBioLab+ software (v.1.5.9, OT Bioelettronica). All HD sEMG signals were amplified using a gain of 1000.

### 2.6 High‐density sEMG analysis

#### 2.6.1 High‐density sEMG: MU decomposition

The HD sEMG signals were decomposed offline using the *Decomponi* feature of the OT BioLab+ software (v.1.5.9, OT Bioelettronica), which is based on convolution blind‐source separation (Negro, Muceli et al., 2016).

The estimated MU spike trains were assessed against a silhouette measure (SIL), which represents the silhouette of the detected MU and was used as a normalized index of reliability (Negro, Muceli et al., 2016). The SIL was set initially at 0.90, then reduced to 0.85 to increase MU yield. Owing to use of a lower SIL value (0.85) to discriminate MUs, additional inclusion criteria for MU acceptance were implemented (Feeney et al., 2018). Only MUs that met the following criteria were accepted for further analysis: (1) pulse‐to‐noise ratio ≥ 28 dB; (2) a mean interspike interval (ISI) between 20 and 200 ms; (3) a skewness for the ISI distribution of less than two; (4) coefficient of variation (CV) of ISI (CV of ISI) of <30%; and (v) an observable waveform in bipolar differential recordings (Feeney et al., 2018). A single experienced investigator inspected all decomposition results visually and excluded all erroneous MUs and discharge times (del Vecchio et al., 2020; Martinez‐Valdes et al., 2023).

The binary representations of the MU spike trains for all isometric KE contractions at 20% of MVC performed by each participant were exported from the OT BioLab+ software (v.1.5.9, OT Bioelettronica) for analysis offline in MATLAB (R2023a; The MathWorks). Before further analysis of the binary MU spike trains, the steadiest 10 s epoch of muscle torque time series from each 20 s isometric KE contraction was identified as the epoch with the lowest SD (determined using a 10 s moving window with a 90% overlap). The binary MU discharges from the same 10 s epoch were extracted for further analysis. The mean discharge rate (DR), DR variability (CV of ISI) and the mean ISI were obtained from the 10 s epoch of all isometric KE contractions at 20% of MVC.

Fifty‐eight participants provided at least six MUs during each of the three fresh isometric KE contractions at 20% of MVC. However, only 36 of the 60 participants provided the minimum number of MUs (at least six MUs) required for within‐muscle coherence analysis during the fatigued isometric KE contractions at 20% of MVC (Dideriksen et al., 2018). As such, data from only 36 participants were used in all further analysis of the study.

The MUs decomposed during the fresh and fatigued isometric KE contractions at 20% of MVC were potentially different, owing to increased MU recruitment during the fatigued contraction. However, like Castronovo et al. (2015), MUs were not tracked across contractions, because it was demonstrated that coherence estimates were not associated with differences in recruitment threshold.

#### 2.6.2 Estimation of within‐muscle MU coherence

Within‐muscle (intra‐muscle) frequency‐domain coherence analysis was used to assess the neural connectivity between MUs of the VL muscle (Myers et al., 2004). Higher coherence values, for the same number of MUs involved in the coherence estimation, indicates a greater strength of common synaptic input to the motoneuron pool (Negro & Farina, 2011). To estimate within‐muscle coherence, the magnitude‐squared coherence was calculated using Welch's averaged periodogram method, with 1 s non‐overlapping windows at different frequency bands: delta (0–5 Hz), alpha (5–15 Hz), low beta (15–21 Hz), high beta (21–35 Hz) and piper (35–50 Hz; Dideriksen et al., 2018). The coherence analysis was repeated for 100 random permutations of two equally sized unfiltered cumulative MU spike trains (CSTs) of unique combinations of three MUs. The averaged value of coherence was computed from all permutations (Hug et al., 2021; Negro, Yavuz et al., 2016).

Following standard practice, coherence estimates were transformed into standard *Z*‐scores using Equation (1) below, where COH is the raw coherence value, *L* is the number of time segments used in the coherence analysis (e.g., for 10 s, *L *= 10, because the analysis was performed on a 10 s window), atanh returns the inverse hyperbolic tangent of the square root of COH, and the bias is calculated empirically as the mean *Z*‐score between 100 and 500 Hz where no coherence is expected.

(1) $$Z=\sqrt{2L}\;\times a\tan h\left(\sqrt{\left(\mathrm{COH}\right)}\right)-\mathrm{bias}$$

The standard *Z*‐transform is an empirical method for removing bias from the signals that are uncorrelated within the specified range (del Vecchio et al., 2019; Rossato et al., 2022). The average *Z*‐score was extracted from each coherence profile within each frequency band. The transformed coherence values can be considered significantly greater than zero at a value of 1.65 (one‐sided 95% confidence level).

### 2.7 Torque signal complexity and magnitude‐metric analysis

The most accurate fresh isometric KE contraction at 20% of MVC, as determined by the contraction with the lowest root mean square error (RMSE), was used for comparison to the fatigued isometric KE contraction at 20% of MVC. Prior to calculation of the KE muscle torque complexity and magnitude‐based metrics, the steadiest 10 s of fresh and fatigued isometric KE contraction at 20% of MVC was identified as the 10 s with the lowest SD (Pethick et al., 2015).

#### 2.7.1 Multiscale entropy analysis

The MSE analysis of the torque signal was performed as outlined by Costa et al. (2002), providing a measure of complexity of the signal over multiple time scales. The MSE analysis overcomes limitations of sample entropy (SampEn), which measures the regularity of time series data on only one time scale, and therefore does not capture the temporal structure of the time series.

The SampEn quantifies the conditional probability that a template length of *m* and *m *+ 1 data points is repeated during the time series within a tolerance of *r* (set at a percentage of the time series SD). Values of SampEn close to zero indicate greater regularity and high predictability in a physiological signal, whereas a value closer to two indicates greater irregularity.

From the one‐dimensional discrete time series, {χ_1_, …, χ*_i_*, …, χ*_n_*}, a coarse‐grained time series was constructed, {*y* ^(τ)^}, determined by the scale factor, τ, according to Equation (2), where τ is the the scale factor, *j* is the time index, *N* is the number of data points, and *i* is the index variable used to iterate through the data points.

(2) $$y\;\begin{array}{c} \left(\tau \right)\\ j \end{array}=\frac{1}{\tau }\;\sum_{i-\left(j-1\right)\tau +1}^{j\tau }\chi_{i^{\prime }}\;1\leq \;j\;\leq N/\tau \;$$

At one scale, the time series {*y* ^(1)^} is the original time series of 10,000 data points (10 s torque epoch). The length of the coarse‐grained time series is equal to the length of the original time series divided by the scale factor, τ, with each coarse‐grained time series capturing the structural and dynamic behaviour of the torque signal at different time scales (i.e., capturing the regularity or randomness of the different oscillation frequencies present in the torque signal). In the present study, the torque time series were coarse‐grained up to scale 28. This was to ensure that the shortest torque time series contained 357 data points, meeting the standards for obtaining a reliable estimate of SampEn (Costa et al., 2002).

The SampEn for each coarse‐grained time series is calculated and plotted against the scale factor, τ, producing an MSE curve. The SampEn of each coarse‐grained torque signal was computed using Equation (3), where *N* is the number of data points in the time series, *m* is the length of the template, *Ai* is the number of matches of the *i*th template of length *m *+ 1 data points, and *Bi* is the number of matches of the *i*th template of length *m* data points. In the present study, template length was set at *m *= 2 and tolerance set at *r* = 10% of the SD of the torque signal.

(3) $$\mathrm{SampEn}\;\left(m,r,N\right)=-\log\;\left(\frac{\sum \begin{array}{c} \;N\;-m\;\\ \;i\;=\;1\; \end{array}A_{i}}{\sum \begin{array}{c} N\;-\;m\\ i\;=\;1 \end{array}B_{i}}\right)=\;-\log\;\left(\begin{array}{c} A\\ B \end{array}\right)$$

The areas under the MSE curve were calculated from scales 1 to 28 using Equation (4):

(4) $$\mathrm{CI}\;=\;\sum_{i\;=\;1}^{\tau }\mathrm{SampEn}\left(i\right)$$

The area under the MSE curve is defined as the complexity index (CI), with higher CI values indicating greater complexity of the physiological signal. The area under all scales of the MSE curve (1–28) was calculated and termed the CI‐28 metric.

#### 2.7.2 Detrended fluctuation analysis

The DFA algorithm was used, as outlined by Peng et al. (1994), to measure the fractal scaling of the torque time series. The DFA algorithm allows for the detection of long‐range correlations embedded in seemingly non‐stationary physiological time‐series data. The torque time series (denoted as *Tor*) is first integrated, using Equation (5):

(5) $$y\left(k\right)\;=\;\sum_{i\;=\;1}^{k}\left(Tor_{i\;}-\;\bar{Tor}\right),\;k\;=\;1,\;\text{…},N$$

The integrated time series are then divided into boxes of equal length, *n*. Within each box length *n*, a straight line is fitted to the data using least squares, denoting the local trend in each box, *y_n_* (*k*). The integrated time series *y*(*k*) is then detrended by subtracting the local trend, *y_n_* (*k*), within each box. The root‐mean‐square fluctuation of the integrated and detrended time series is calculated by Equation (6):

(6) $$F\left(n\right)=\sqrt{\frac{1}{N}\sum_{k=1}^{N}{\left[y\left(k\right)-y_{n}\left(k\right)\right]}^{2}}$$

The DFA computation (6) is repeated across all box sizes to provide a relationship between *F*(*n)*, the average fluctuation as a function of box size, and the box size, *n*, the number of data points in a box (Peng et al., 1995). The slope of the double logarithmic plot, log *F*(*n*) versus log *n*, determines the scaling exponent, α. DFA α was calculated with 57 box sizes ranging from 1250 to 4 data points as recommended by Pethick et al. (2015).

The DFA produces a scaling exponent, α. A value of α = 0.5 indicates that one value of the time series is completely uncorrelated from any previous values (i.e., unpredictable white noise; indicative of a very rough time series). A value of α = 1.5 indicates brown noise (integral of white noise) and a loss of long‐range correlations (i.e., a smooth output with long‐term memory). A perfectly persistent physiological time series would have a scaling exponent α of 1.0 (i.e., 1/*f* or pink noise), suggestive of a physiological output of high complexity, i.e., statistically self‐similar with long‐range correlations. A value of α between 0.5 and 1 indicates persistent long‐range power‐law correlations, such that a large number is more likely to be followed by a large number and vice versa. A value of α between 1 and 1.5 indicates that correlations exist but are not power‐law form (Peng et al., 1995).

#### 2.7.3 Magnitude‐based metric analysis

The absolute variability of the torque output of participants was quantified using the standard deviation of torque (SDT), and the CVT [CV = (SD/mean torque) × 100] was calculated to quantify the variability in the torque output of participants normalized to the mean of the torque output. The accuracy of torque was quantified by calculating the RMSE between the target torque and the instantaneous torque during the steadiest 10 s of the isometric contraction.

### 2.8 Cross‐correlation analysis of torque and effective neural drive

The individual binary MU spike trains from the steadiest 10 s epoch of the isometric contraction were summed to produce a CST. To extract the low‐frequency components of effective neural drive to the muscle, which are representative of torque fluctuation dynamics, the CSTs were smoothed with a 400 ms Hann window, and high‐pass filtered (0.75 Hz, second order zero‐lag, Butterworth) to remove trends and isolate the fluctuations in neural drive (Mazzo et al., 2022; Negro et al., 2009; Thompson et al., 2018). The torque signal was low‐pass filtered (20 Hz, fourth order, Butterworth) and high‐pass filtered (0.75 Hz, second order zero‐lag, Butterworth) for comparison with the estimates of neural drive. The cross‐correlation (time domain) between the estimates of effective neural drive (the CST) and isometric KE torque was calculated across the entire 10 s contraction (Mazzo et al., 2022; Negro et al., 2009; Thompson et al., 2018).

### 2.9 Data analysis

All torque and processed HD sEMG data were analysed using code written in MATLAB R2023a (R2023a; The MathWorks).

### 2.10 Statistical analysis

The effect of age (YG, MG and OG) on TTF was assessed with a one‐way ANOVA.

The effect of condition (fresh vs. fatigued) on the within‐muscle coherence *Z*‐scores was assessed using a mixed ANOVA, with condition (fresh vs. fatigued) and frequency band (delta, alpha, low beta, high beta and piper) as within‐subject factors and with age groups (YG, MG and OG) as the between‐subject factors.

The effect of condition on the MSE analysis of the torque signals was assessed using a mixed ANOVA, with condition (fresh vs. fatigued) and coarse‐grained scale (1–28) as within‐subject factors and with age groups (YG, MG and OG) as the between‐subject factors.

The effect of condition (fresh vs. fatigued) and age group (YG, MG and OG) was assessed using a two‐way ANOVA to determine between‐ and within‐condition effects on all MU characteristics and all torque control metrics.

The relationship between participant age, MVC, absolute intensity at 20% of MVC, coherence *Z*‐scores at each frequency band (delta, alpha, low beta, high beta and piper), the MU characteristics (CV of ISI, DR and number of MUs) and the torque control metrics (DFA, MSE, CI‐28, CVT, SDT and RMSE) were established using Pearson's bivariate two‐tailed correlation. Following this, a hierarchical, multiple, linear regression analysis was used to assess the predictive capacity of the alpha band coherence *Z*‐scores on the torque control metrics (MSE, DFA, CI‐28, CVT, SDT and RMSE), with participant age and the absolute intensity at 20% of MVC held constant.

Hierarchical, multiple, linear regression analysis was used to assess the predictive capacity of the fatigue‐related change in the delta, alpha and low beta band coherence *Z*‐scores on the fatigue‐related change in the torque control metrics (MSE, DFA, CI‐28, CVT, SDT and RMSE), with participant age and TTF held constant.

Statistical assumptions of linearity, multicollinearity, additivity, homoscedasticity, independence of residuals and normality of residuals were checked and accepted unless otherwise stated. Partial eta squared (η_p_^2^; interpreted as: 0.01 small effect, 0.06 medium effect and 0.14 large effect) were used to assess effect sizes. Bonferroni *post hoc* comparisons were used when a main effect or interaction was significant. The significance level was set at *P* < 0.05 in all cases. Statistical analyses were performed in IBM SPSS statistics v.29 (IBM Corp., Armonk, NY, USA).

## 3 RESULTS

### 3.1 Preliminary measures

Of the 60 participants, 36 provided enough MUs (at least six MUs) required for within‐muscle coherence analysis during both the fresh and fatigued isometric KE at 20% of MVC. As such, only data from these 36 participants were used in all further analysis. Table 1 presents the participant characteristics of the 36 participants.

**TABLE 1:** Participant characteristics.

Characteristic	All participants	Younger group	Middle group	Older group
*n*	36 (35 male, 1 female)	9 (9 male, 0 female)	14 (13 male, 1 female)	13 (13 male, 0 female)
Age, years	44.3 ± 15.4	25.8 ± 3.7	41.0 ± 4.2	60.7 ± 10.3
Height, cm	177.6 ± 7.0	180.2 ± 2.9	176.5 ± 9.0	176.8 ± 6.5
Mass, kg	74.2 ± 10.3	69.1 ± 7.9	73.5 ± 10.8	78.7 ± 10.0
VL skinfold, mm	7.0 ± 3.1	7.3 ± 3.7	7.7 ± 2.5	6.1 ± 3.1
MVC, N m	184.6 ± 61.6	236.9 ± 62.7	185.4 ± 61.3	147.6 ± 28.9
20% MVC, N m	36.9 ± 12.3	47.4 ± 12.5	37.1 ± 12.3	29.5 ± 5.8
60% MVC, N m	110.8 ± 37.0	142.2 ± 37.6	111.2 ± 36.8	88.6 ± 17.3

*Note*: Data are the means ± SD. Abbreviations: MVC, maximal voluntary contraction; VL, vastus lateralis muscle.

The TTF was significantly longer for the OG (399.6 ± 116.1 s), in comparison to the YG (273.9 ± 157.2 s; *P *= 0.0112). There was no significant difference in TTF between the MG (310.4 ± 114.1 s) and OG (*P *= 0.201) or YG (*P *= 1.000).

### 3.2 Motor unit characteristics

Table 2 presents the MU characteristics of the 36 participants who provided at least six MUs during both the fresh and fatigued isometric KE at 20% of MVC. There was no difference in the number of MUs decomposed and accepted from the fresh and fatigued isometric KE at 20% of MVC (*P *= 0.161; Table 2).

**TABLE 2:** Motor unit characteristics with ANOVA results.

Characteristic	Group				*F*, *P* and η_p_^2^ values		
		Fresh 20% of MVC	Fatigued 20% of MVC		Condition	Age group	Condition by age
Total MU, *n*	All YG MG OG	330 73 118 139	314 65 112 137				
MU per contraction, *n*	All YG MG OG	9.17 ± 3.31 8.11 ± 1.36 8.43 ± 2.71 10.69 ± 4.33	8.72 ± 3.29 7.22 ± 1.30 8.00 ± 2.29 10.54 ± 4.35	*F* *P* η_p_^2^	2.055 0.161 0.059	3.344 0.0476 0.169	0.354 0.704 0.021
DR, pulses/s	All YG MG OG	8.80 ± 1.61 9.88 ± 1.49 9.01 ± 1.49 7.82 ± 1.32	8.82 ± 1.74 10.35 ± 1.82 8.85 ± 1.42 7.73 ± 1.16	*F* *P* η_p_^2^	0.090 0.766 0.003	9.515 0.000546 0.366	0.588 0.561 0.034
Mean ISI, ms	All YG MG OG	114.17 ± 17.74 102.08 ± 10.06 111.08 ± 17.12 125.88 ± 16.23	121.78 ± 22.86 104.09 ± 19.34 119.10 ± 18.81 136.90 ± 20.01	*F* *P* η_p_^2^	4.583 0.0398 0.122	10.469 0.000301 0.388	0.585 0.563 0.034
CV of ISI, %	All YG MG OG	19.63 ± 3.45 21.10 ± 4.12 19.82 ± 2.39 18.42 ± 3.72	20.87 ± 4.12 23.49 ± 2.58 21.32 ± 3.68 18.56 ± 4.39	*F* *P* η_p_^2^	5.368 0.0269 0.140	4.060 0.0265 0.197	1.227 0.306 0.069

*Note*: Data are the mean ± SD. Abbreviations: CV, coefficient of variation; DR, discharge rate; ISI, interspike interval; MG, middle group; OG, older group; MU, motor unit; MVC, maximal voluntary contraction; PPS, pulses per second; YG, younger group.

### 3.3 Motor unit within‐muscle coherence results

**FIGURE 2:**
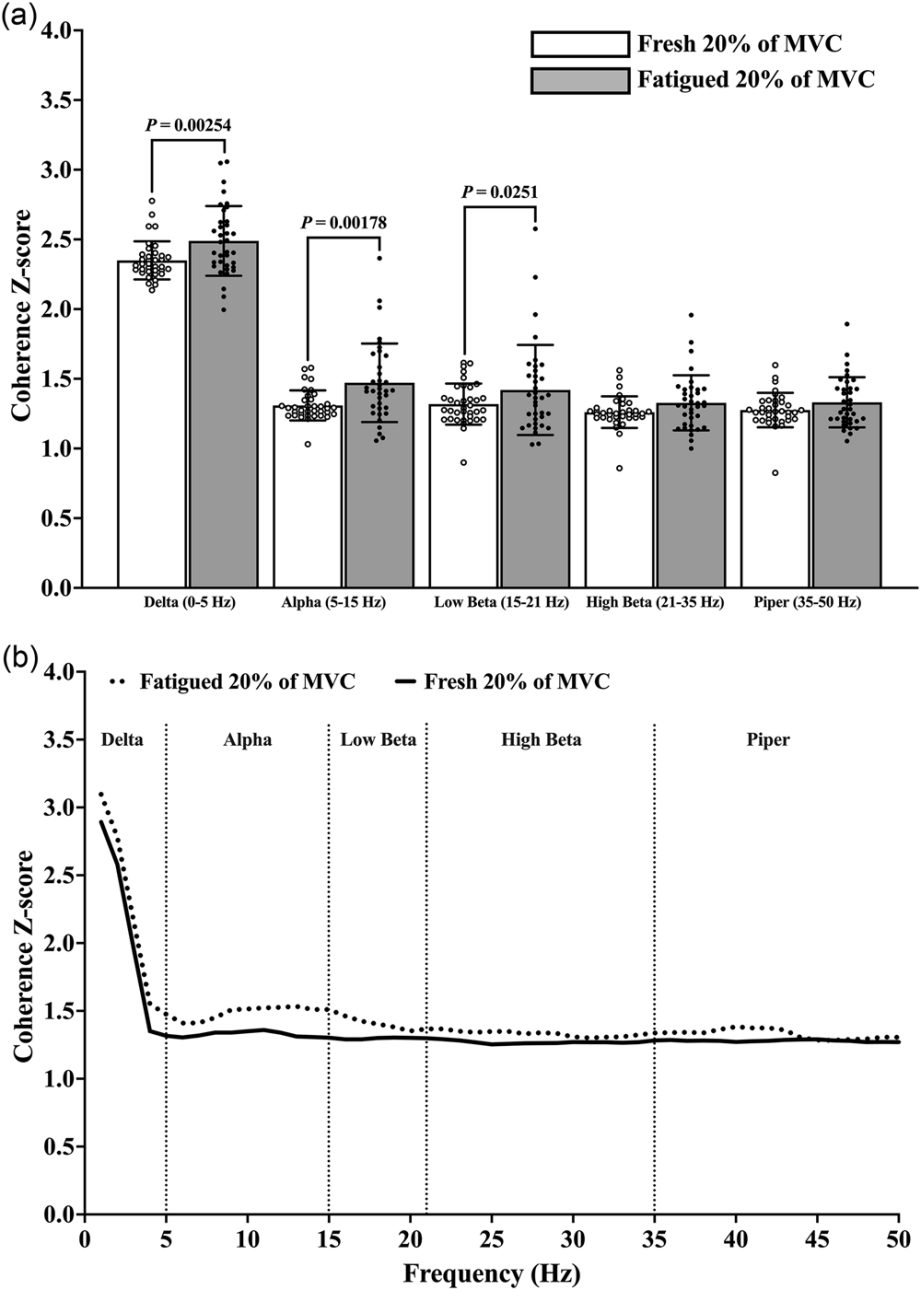
(a) Within‐muscle coherence *Z*‐scores at the different frequency bands during the fresh (open circles and columns) and fatigued (filled circles and columns) isometric KE contractions at 20% of MVC; (b) The group mean coherence *Z*‐scores from 0 to 50 Hz of the fresh (continuous line) and fatigued (dotted line) isometric KE contractions at 20% of MVC. Abbreviations: KE, knee extensor; MVC, maximal voluntary contraction.

There was a significant main effect of condition (fresh vs. fatigued) on within‐muscle coherence *Z*‐scores (*F*_1,333_ = 11.603; *P *= 0.00175; η_p_^2^ = 0.260), indicating that overall, coherence *Z*‐scores (all frequency bands) were higher during the fatigued contractions in comparison to the fresh contractions. *Post hoc* pairwise comparisons revealed the delta (*P *= 0.00254), alpha (*P* = 0.00178) and low beta (*P *= 0.0251) frequency band coherence *Z*‐scores to be significantly higher during the fatigued contraction in comparison to the fresh contractions (Figure 2).

There was no main effect of age group on within‐muscle coherence (*F*_2,33_ = 0.473; *P *= 0.627; η_p_^2^ = 0.028), indicating that overall, coherence *Z*‐scores (all frequency bands) were not different between the YG, MG and OG. There was no condition (fresh vs. fatigued) by age group interaction (*F*_2,33_ = 1.443; *P *= 0.2507; η_p_^2^ = 0.080), indicating that overall, the effect of age group on the coherence *Z*‐scores was consistent across both the fresh and fatigued conditions.

### 3.4 Knee extensor torque control results

#### 3.4.1 Multiscale entropy results

There was no main effect of condition (fresh vs. fatigued; *F*_1,33_ = 1.949; *P *= 0.172; η_p_^2^ = 0.056), no main effect of age group (*F*_2,33_ = 1.000; *P *= 0.379; η_p_^2^ = 0.057) and no condition by age group interaction (*F*_2,33_ = 0.613; *P *= 0.548; η_p_^2^ = 0.036) for SampEn calculated across the 28 coarse‐grained scales of the MSE curve.

**FIGURE 3:**
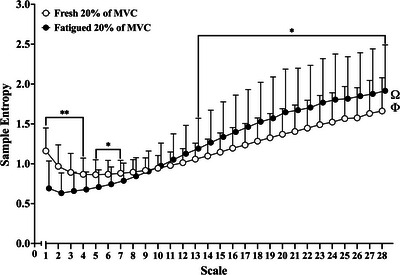
Multiscale entropy curve of sample entropy derived from the torque signals of the fresh and fatigued isometric KE at 20% of MVC (open circles, fresh 20% of MVC; filled circles, fatigued 20% of MVC; Ω, significant main effect of scale; Φ, significant condition by scale interaction; *P < 0.05; **P < 0.0001; all exact *post hoc P*‐values for comparisons at each coarse‐grained scale can be found in the Supplementary material). Abbreviations: KE, knee extensor; MVC, maximal voluntary contraction.

There was a main effect of coarse‐grained scale (*F*_27,891_ = 189.126; *P *< 0.0001; η_p_^2^ = 0.851) and a significant condition (fresh vs. fatigued) by coarse‐grained scale interaction (*F*_27,891_ = 38.722; *P *< 0.0001; η_p_^2^ = 0.540). *Post hoc* pairwise comparisons revealed that at shorter scales (≤scale 7) the fresh contractions exhibited significantly higher SampEn, in comparison to the fatigued contractions (all *P *< 0.05; Figure 3). At longer (coarser) scales (≥scale 13), the fatigued contractions exhibited significantly higher SampEn, in comparison to the fresh contractions (all *P *< 0.05; Figure 3).

**FIGURE 4:**
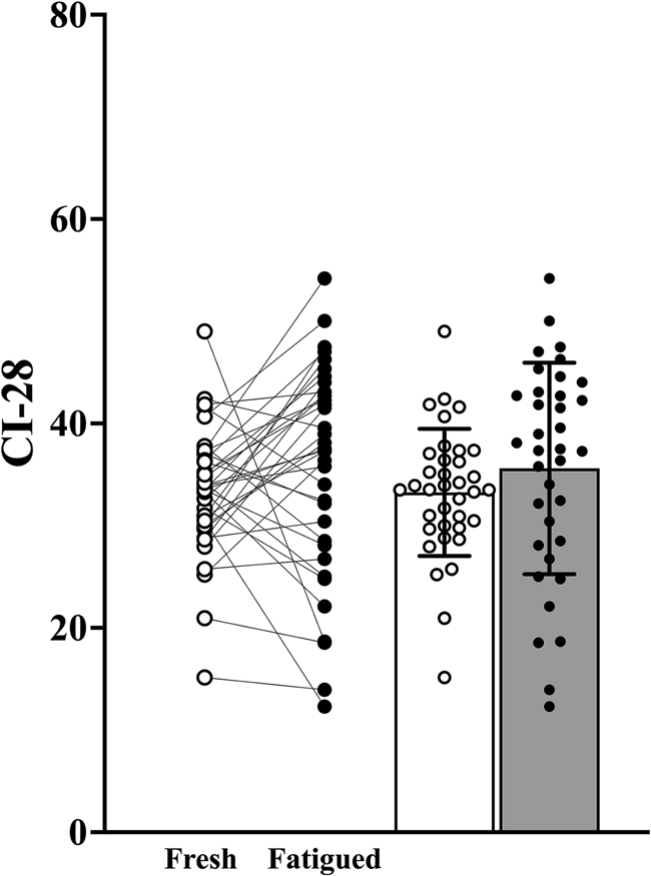
Before–after and bar plots of the complexity index under 28 coarse‐grained scales (CI‐28) derived from the torque signals of the fresh and fatigued isometric KE at 20% of MVC (open circles and columns, fresh 20% of MVC; filled circles and columns, fatigued 20% of MVC). Abbreviations: KE, knee extensor; MVC, maximal voluntary contraction.

There was no main effect of condition (fresh vs. fatigued; *F*_1,33_ = 1.950; *P *= 0.172; η_p_^2^ = 0.056), no main effect of age group (*F*_2,33_ = 1.000; *P *= 0.379; η_p_^2^ = 0.057) and no significant condition by age group interaction (*F*_2,33_ = 0.613; *P *= 0.548; η_p_^2^ = 0.036) for the CI‐28 metric (Figure 4).

#### 3.4.2 Detrended fluctuation analysis results

**FIGURE 5:**
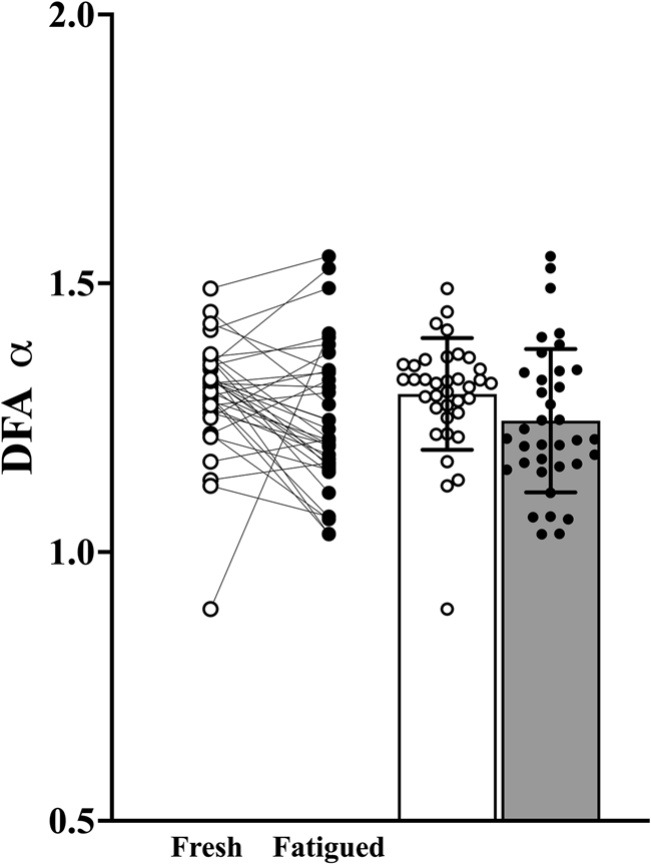
Before–after and bar plots of the detrended fluctuation analysis scaling exponent α (DFA α) derived from the torque signals of the fresh and fatigued isometric KE at 20% of MVC (open circles and columns, fresh 20% of MVC; filled circles and columns, fatigued 20% of MVC). Abbreviations: KE, knee extensor; MVC, maximal voluntary contraction.

There was no main effect of condition (fresh vs. fatigued; *F*_1,33_ = 3.560; *P *= 0.0680; η_p_^2^ = 0.097), no main effect of age group (*F*_2,33_ = 0.783; *P *= 0.465; η_p_^2^ = 0.045) and no significant condition by age group interaction (*F*_2,33_ = 0.207; *P *= 0.814; η_p_^2^ = 0.012) for the DFA α metric (Figure 5).

#### 3.4.3 Magnitude‐based metric results

**FIGURE 6:**
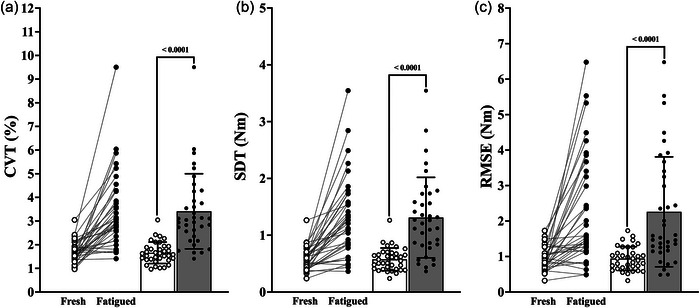
Before–after and bar plots of the magnitude metrics derived from the torque signals of the fresh and fatigued isometric KE at 20% of MVC. (a) Coefficient of variation of torque (CVT). (b) Standard deviation of torque (SDT). (c) Root mean squared error (RMSE) of torque (open circles and columns, fresh 20% of MVC; filled circles and columns, fatigued 20% of MVC). Abbreviations: KE, knee extensor; MVC, maximal voluntary contraction.

The CVT (main effect of condition, *F*_1,33_ = 43.331; *P *< 0.0001; η_p_^2^ = 0.568; Figure 6a), SDT (main effect of condition, *F*_1,33_ = 45.041; *P *< 0.0001; η_p_^2^ = 0.577; Figure 6b) and RMSE of torque (main effect of condition, *F*_1,33_ = 24.837; *P *< 0.0001; η_p_^2^ = 0.429; Figure 6c) were significantly higher during fatigued isometric KE at 20% of MVC in comparison to the fresh isometric KE at 20% of MVC.

There was no effect of age group on the CVT (*F*_2,33_ = 0.292; *P *= 0.749; η_p_^2^ = 0.017), the SDT (*F*_2,33_ = 2.031; *P *= 0.147; η_p_^2^ = 0.110) and the RMSE of torque (*F*_2,33_ = 1.883; *P *= 0.168; η_p_^2^ = 0.102). There was no condition by age group interaction for the CVT (*F*_2,33_ = 0.833; *P *= 0.444; η_p_^2^ = 0.048), the SDT (*F*_2,33 _= 1.579; *P *= 0.221; η_p_^2^ = 0.087) and the RMSE of torque (*F*_2,33_ = 1.529; *P *= 0.232; η_p_^2^ = 0.085).

### 3.5 Relationship between within‐muscle coherence and the KE torque control metrics

**FIGURE 7:**
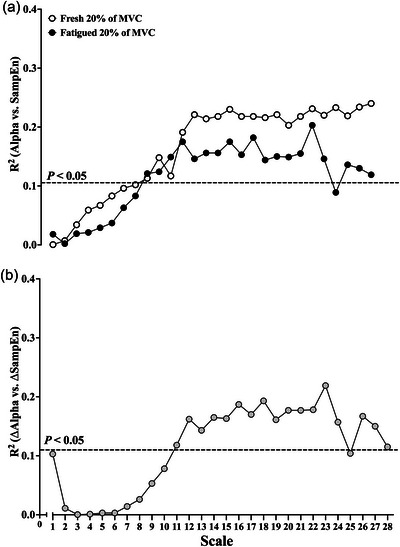
(a) Relationship (*R* ^2^ values) between the alpha band coherence *Z*‐scores and the sample entropy at each coarse‐grained scale of the MSE derived from the fresh and fatigued isometric KE at 20% of MVC. (b) Relationship (*R* ^2^ values) between the fatigued‐related change in alpha band coherence *Z*‐scores and the fatigue‐related change in sample entropy at each coarse‐grained scale of the MSE (open circles, fresh 20% of MVC; filled circles, fatigued 20% of MVC; grey circles, Δalpha vs. ΔSampEn; dashed line, significant correlation at *P *< 0.05; all exact *P* and *R* ^2^ values for comparisons at each coarse‐grained scale can be found in the Supplementary material). Abbreviations: KE, knee extensor; MSE, multiscale entropy analysis; MVC, maximal voluntary contraction.

The hierarchical, multiple, linear regression analysis revealed alpha band coherence *Z*‐scores to be significantly associated with SampEn of the coarse‐grained torque signal from scales 9 to 28 during both the fresh and fatigued isometric KE at 20% of MVC (all *P *< 0.05; Figure 7a).

Alpha band coherence *Z*‐scores were not significantly predictive of SampEn at scale 1 (i.e., SampEn of the original torque signal) during both the fresh (*P = *0.883) and fatigued (*P = *0.399) isometric KE at 20% of MVC (Table 3; Figure 7a). In contrast, alpha band coherence explained 24.0% of the variance in SampEn at scale 28 during the fresh KE contractions (Table 3; Figure 7a) and explained 11.9% of the variance in SampEn at scale 28 during the fatigued KE contractions (Table 3; Figure 7a).

**TABLE 3:** Statistics from the hierarchical, multiple, linear regression analyses of the torque control metrics.

Conditions	Dependent variables		Predictor variable: Alpha band coherence		
		*R* ^2^	β Coefficient	*P*‐value	Δ*F*
Fresh 20% of MVC	DFA α CI‐28 SampEn scale 1 SampEn scale 28 CVT SDT RMSE	0.314 0.231 <0.001 0.240 0.004 0.004 0.001	−0.568 0.487 −0.0210 0.497 0.020 0.064 0.026	**<0.0001** **0.00242** 0.883 **0.00104** 0.901 0.633 0.868	14.938 10.850 0.0220 13.001 0.016 0.232 0.028
Fatigued 20% of MVC	DFA α CI‐28 SampEn scale 1 SampEn scale 28 CVT SDT RMSE	0.120 0.136 0.018 0.119 0.150 0.132 0.064	−0.361 0.384 −0.140 0.360 0.403 0.378 0.264	**0.0361** **0.0192** 0.399 **0.0273** **0.0236** **0.0164** 0.1340	4.787 6.080 0.731 5.353 5.653 6.422 2.367
	Dependent variables	Predictor variable: Fatigue‐related Δalpha band coherence			
		*R* ^2^	β Coefficient	*P*‐value	Δ*F*
Fatigue‐related change	ΔDFA α ΔCI‐28 ΔSampEn scale 1 ΔSampEn scale 28 ΔCVT ΔSDT ΔRMSE	0.094 0.118 0.103 0.115 0.190 0.217 0.154	−0.322 0.362 −0.337 0.356 0.458 0.491 0.414	**0.0491** **0.0400** 0.0643 **0.0442** 0.00859 **0.00386** **0.0214**	4.184 4.584 3.672 4.388 7.839 9.705 5.849

Abbreviations: CI‐28, complexity index under 28 coarse‐grained scales of the torque multiscale entropy curves; CVT, coefficient of variation of torque; DFA α, detrended fluctuation analysis scaling exponent α; MVC, maximal voluntary contraction of the knee extensors; RMSE, root mean square error of torque; SDT, standard deviation of torque.

The fatigue‐related Δalpha band coherence *Z*‐scores were significantly predictive of the fatigue‐related ΔSampEn of the coarse‐grained torque signal from scales 11 to 28 (all *P *< 0.05; Figure 7b). The fatigue‐related Δalpha band coherence *Z*‐scores were not significantly predictive of fatigue‐related ΔSampEn at scale 1 (Table 3; Figure 7b). In contrast, the fatigue‐related Δalpha band coherence explained 11.5% of the variance in the fatigue‐related ΔSampEn at scale 28 (Table 3; Figure 7b).

Alpha band coherence explained 23.1% and 13.6% of the variance in the CI‐28 torque metric during the fresh and fatigued KE contractions, respectively (Table 3). The fatigue‐related Δalpha band coherence explained 11.8% of the variance in the fatigue‐related ΔCI‐28 torque metric (Table 3).

Alpha band coherence explained 31.4% and 12.0% of the variance in DFA α of the torque signal during the fresh and fatigued KE contractions, respectively (Table 3). The fatigue‐related Δalpha band coherence explained 9.4% of the variance in the fatigue‐related ΔDFA α (Table 3).

The fatigued‐related Δalpha band coherence could predict 19.0%, 21.7% and 15.4% of the variance in the fatigued‐related ΔCVT, ΔSDT and ΔRMSE, respectively (Table 3).

Alpha band coherence could explain 15.0% and 13.2% of the variance in the CVT and SDT, respectively, during the fatigued KE contractions at 20% of MVC. In comparison, alpha band coherence was not significantly predictive of the CVT or SDT during the fresh KE contractions at 20% of MVC (Table 3).

### 3.6 Cross‐correlation results of torque and effective neural drive

**FIGURE 8:**
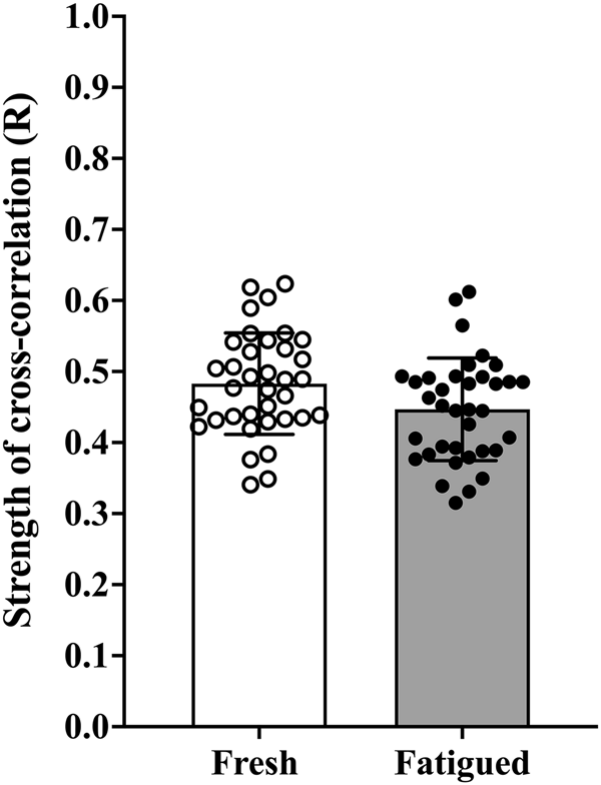
Cross‐correlation coefficients for the estimate of effective neural drive and the fluctuations in the fresh and fatigued isometric knee extensor torque signals (open circles and column, fresh 20% of MVC; filled circles and column, fatigued 20% of MVC). Abbreviation: MVC, maximal voluntary contraction.

The estimate of effective neural drive was moderately correlated with the fluctuations in the fresh and fatigued isometric KE torque signals (Figure 8).

## 4 DISCUSSION

In the present study, we investigated whether the strength of oscillations in common synaptic input, assessed by the within‐muscle coherence of CSTs, was explanatory of KE torque signal complexity during fresh and fatigued submaximal isometric contractions, in adults aged from 18 to 90 years. The new findings were that alpha band coherence (5–15 Hz) was significantly related to the torque complexity metrics CI‐28, DFA α (Table 3) and SampEn at scales 9–28 of the MSE curves (Figure 7a), during the fresh isometric KE at 20% of MVC. However, in contrast to the hypothesis of previous researchers (Pethick et al., 2021b, 2016), participants with higher alpha band coherence during the fresh isometric contractions exhibited KE torque signals of higher complexity (i.e., a higher CI‐28 and a DFA α closer to 1.0) and greater irregularity (i.e., a higher SampEn).

The fatiguing isometric KE protocol resulted in an increase in the strength of common synaptic input, with significant increases in the delta (common drive band), alpha (physiological tremor band) and low beta frequency bands (Figure 2a). There was a significant fatigue‐related increase in torque signal variability (Figure 6), but no significant fatigue‐related change in the CI‐28 (Figure 4) and DFA α (Figure 5) complexity metrics. The fatigue‐related change in alpha band coherence was significantly predictive of the fatigue‐related changes in the CVT, SDT, RMSE (Table 3) and the SampEn of the coarse‐grained torque signal from scales 11 to 28 of the MSE curve (Figure 7b).

Contrary to prior hypotheses, the present findings indicate that greater fatigue‐related increases in alpha band coherence are likely to be accompanied by larger increases in torque signal complexity (i.e., increase in CI‐28 and decrease in DFA α) and signal irregularity (i.e., increase in SampEn). However, in accordance with our hypothesis, participants who demonstrated greater fatigue‐related increases in alpha band coherence were also likely to exhibit larger increases in the CVT, SDT and RMSE.

### 4.1 Relationship between the strength of oscillations in common synaptic input and fresh isometric KE torque complexity

This study presents the first evidence establishing a relationship between the strength of oscillations in common synaptic input and the complexity measures used to analyse the temporal structure of torque signals during isometric contractions. Specifically, when controlling for age, the strength of alpha (physiological tremor) band oscillations in common synaptic input was found to predict 23.1% and 31.4% of the variance in the CI‐28 and DFA α complexity metrics, respectively (Table 3). Additionally, the strength of alpha band oscillations was found to predict between 11.3% and 24.0% of the variance in SampEn from scales 9 to 28 of the MSE curves derived from the fresh contractions at 20% of MVC (Figure 7a). The involuntary oscillations in the alpha frequency band are associated with physiological tremor (Lippold, 1971) and can be attributed, in part, to proprioceptive (Ia) afferent feedback from the active muscles that project to the motoneuron pool (Lippold, 1970; Mehrkanoon et al., 2014). Therefore, it can be inferred that the complexity of a submaximal isometric KE torque signal might be indicative of the amount of Ia afferent feedback and the ability to modulate Ia presynaptic inhibition.

The absence of a relationship between the delta frequency band (0–5 Hz) and the torque complexity metrics is notable, particularly considering that low‐frequency components (<10 Hz) of neural drive are typically transformed into muscle torque (Farina & Negro, 2015). This might imply that these complexity metrics (MSE, DFA and CI‐28) are unable to capture the temporal structure of the torque signal produced by common synaptic input at <5 Hz.

The alpha frequency band (5–15 Hz) encompasses frequencies of effective neural drive, in addition to higher‐frequency common inputs (Farina & Negro, 2015). It is conceivable that these higher‐frequency common inputs at or above those of effective neural drive are embedded as common irregularities, through non‐linear amplitude modulation, into the lower‐frequency oscillations in common neural drive (Watanabe & Kohn, 2015). These irregularities in common neural drive might then be transmitted into the muscle torque signal, if the input is common to a sufficient number of motoneurons (Farina & Negro, 2015). This non‐linear transmission might, potentially, explain why stronger alpha frequency band oscillations in common synaptic input are associated with more irregular, higher‐complexity torque signals (Table 3; Figure 7a). Although speculative, the presence of these irregularities in the torque signal suggests that the non‐linear interactions between the different frequency bands of common input might contribute to the complexity in the muscle torque signal. However, the strength of alpha band oscillations could explain only part of the complexity of the torque signal; therefore, further research is required to determine the neural control mechanisms underpinning the unexplained variance in torque signal complexity.

### 4.2 Effect of neuromuscular fatigue on the strength of common synaptic input and torque control

In accordance with existing literature, there was an increase in the strength of common synaptic input when the muscle was fatigued (Castronovo et al., 2015; Contessa et al., 2009; McManus et al., 2016; Rossato et al., 2022). The observed increase in the strength of common input at the higher oscillatory frequencies (alpha and low beta coherence bands; Figure 2) is likely to reflect the reduced inhibition of the Ia afferent feedback loop and increased corticospinal excitation and descending drive as the muscle is fatigued (Castronovo et al., 2015; Mehrkanoon et al., 2014).

Immediately following task failure, participants were able to meet the target torque set at 20% of MVC, albeit with a significantly higher torque variability and greater targeting errors in comparison to fresh contractions of a matched intensity (Figure 6). The observed fatigue‐related increase in alpha band oscillations in common synaptic input could account for 19.0%, 21.7% and 15.4% of the corresponding increases in the CVT, SDT and RMSE, respectively (Table 3). The present findings corroborate earlier research, which linked fatigue‐related increases in the strength of common synaptic input with increases in muscle torque signal variability (Castronovo et al., 2015; Contessa et al., 2009; McManus et al., 2016).

Alpha band oscillations in common synaptic input (common noise) are suggested to be associated with error corrections and overshoots in movement during tasks of muscle torque control (Mehrkanoon et al., 2014). This is likely to explain the concurrent fatigue‐related decrease in muscle torque control and increase in alpha band oscillations, in addition to the significant association between the strength of alpha band oscillations and torque signal variability during the fatigued contractions (Table 3). The absence of a clear relationship between alpha band oscillations and isometric KE torque control during the fresh contractions at 20% of MVC supports this interpretation (Table 3), because participants performed steadier contractions (Figure 6a,b) and made smaller targeting errors (Figure 6c). These findings highlight the involvement of alpha frequency band (physiological tremor band) oscillations in modulating the precision of isometric KE muscle torque output.

There was no significant main effect of fatigue on the complexity metrics CI‐28 (Figure 4) and DFA α (Figure 5), although notable inter‐individual variability in the fatigue‐related changes was evident in both metrics. The fatigue‐related change in the strength of common input in the alpha band accounted for a small yet significant percentage of the inter‐individual variability in the complexity metrics, CI‐28 (11.8%) and DFA α (9.4%). Interestingly, during the fresh contractions, common input in the alpha band explained a greater percentage of the variance in CI‐28 (23.1% for fresh contractions vs. 13.6% for fatigued contractions) and DFA α (31.4% for fresh contractions vs. 12.0% for fatigued contractions) metrics, in comparison to the fatigued contractions (Table 3). The direction and magnitude of change in muscle torque signal complexity attributable to fatigue warrants further investigation, because this appears to be driven primarily by mechanisms other than the observed increases in the strength of common synaptic input in the alpha frequency band.

The present study is the first to assess the temporal structure of isometric KE torque signals using the MSE analysis. Consistent with previous findings (Pethick et al., 2015), fatigue resulted in a significant decrease in SampEn (SampEn at scale 1 of the MSE curves), indicating increased regularity of the original torque signal (Figure 3). However, SampEn at scale 1 fails to describe the scale‐to‐scale alterations in torque signal regularity (the temporal structure of the torque signal), as illustrated by the cross‐over in the MSE curves derived from the fresh and fatigued contractions (Figure 3). During the fatigued contractions, SampEn was lower at shorter scales (≤scale 9) and higher at longer (coarser) scales (≥scale 10) when compared with the fresh contractions (Figure 3).

The cross‐over in SampEn at longer (coarser) scales is noteworthy, because the coarse‐graining procedure progressively reveals the lower‐frequency fluctuations present in the torque signal (Knol et al., 2019; Vaillancourt et al., 2004; Vieluf et al., 2015). The present findings indicate that the fluctuations contained within the torque signals become progressively more irregular and unpredictable at lower frequencies. Importantly, fatigue appears to alter the temporal structure of the torque signals, simultaneously increasing the regularity at shorter scales and decreasing the regularity at longer scales in comparison to the fresh torque signals.

The fatigue‐related decrease in SampEn at coarse‐grained scales 1–9 was not associated with the change in the strength of alpha band oscillations in common synaptic input (Figure 7b). However, from the point of cross‐over in the MSE curves (scales 11–28), the fatigue‐related increase in SampEn was significantly predicted by the increase in the strength of alpha band oscillations in common synaptic input (Figure 7b). Therefore, the greater irregularity of the lower‐frequency fluctuations in the fatigued torque signals might be explained, in part, by the fatigue‐related increase in the strength of higher frequency oscillations in common synaptic input, hence suggesting that higher‐frequency common inputs at or above those of effective neural drive might be embedded as irregularities in the lower‐frequency components of the torque signal and captured as a higher SampEn at the longer (coarser) scales of the MSE curves.

It is notable that there was not a significant association between the fatigue‐related increase in the alpha band oscillations and fatigue‐related decline in SampEn at scale 1 of the MSE curve (Table 3; Figure 7b), particularly given that the hypothesis regarding the association between common synaptic input and the complexity of isometric torque was based, in part, upon the observed fatigue‐related decline in single scale SampEn (Pethick et al., 2015, 2016). The present study is unable to provide an explanation of the mechanisms underpinning SampEn at the shorter coarse‐grained scales (≤scale 9) of the MSE curves. Therefore, research is required to establish the mechanisms that might determine SampEn at shorter scales.

### 4.3 Interaction between age and fatigue on common synaptic input and torque control

This study represents the first assessment of the effect of age on fatigue‐related changes in the estimated strength of common synaptic input and isometric KE torque signal complexity. Age did not emerge as a significant contributory factor to the fatigue‐related changes in the strength of common synaptic input or isometric KE torque signal complexity. It is plausible that the participants of the OG were too young (aged 60.7 ± 10.3 years; Table 1) for us to observe any age‐related factors, including the increased strength of common synaptic input, which might impair muscle control (Mynark & Koceja, 2001; Shaffer & Harrison, 2007). Additionally, an effect of age on torque control might be attenuated during low‐difficulty motor tasks, such as constant isometric contractions (Bootsma et al., 2021; Guo et al., 2024). To elucidate the potential effects of age on fatigue‐related changes in the strength of common synaptic input and torque signal complexity, future studies might need to recruit older participants (>70 or 80 years) and use more complex tasks, such as ramp isometric contractions (Guo et al., 2024).

### 4.4 Cross‐correlation between torque and effective neural drive

The subsidiary aim of the study was to determine the strength of association between the effective neural drive to the VL muscle and fluctuations in isometric KE torque during fresh and fatigued contractions. The estimate of effective neural drive (CST) was found to be moderately correlated with the fluctuations in the fresh and fatigued KE torque signals (Figure 8). This suggests that the spatial structure of the fluctuations in isometric KE muscle torque is determined largely by the low‐frequency oscillations in effective neural drive. The present findings extend previous experimental research using HD sEMG that has demonstrated the fluctuations in finger abduction force, ankle dorsiflexion torque (Negro et al., 2009) and plantar‐flexion torque (Mazzo et al., 2022) to resemble closely the low‐frequency oscillations in effective neural drive.

### 4.5 Limitations

Isometric KE torque is produced through the co‐activation of multiple muscles. However, in the present study, HD sEMG signals were recorded only from VL muscle. The complexity metrics derived from KE torque signal are likely to reflect both the intra‐muscle and inter‐muscle common synaptic input of all KE muscles. In addition, the number of MUs decomposed and accepted from the VL was small in comparison to the number of potentially active MUs, albeit consistent with accepted numbers of MUs in previous research (Dideriksen et al., 2018; Laine et al., 2015). Future research endeavours should aim to record a greater number of MUs from the range of muscles involved in the production of the torque output, to gain further insights into the role of common synaptic inputs in influencing the complexity of muscle torque signals. The intra‐muscle and inter‐muscle common synaptic input to the motoneuron pools of the other KE muscles might also explain additional variance in the complexity metrics, not explained by the common synaptic input to the motoneuron pool of the VL muscle.

Contraction intensity has been shown to influence torque signal complexity, with a loss of isometric KE torque complexity suggested to occur exclusively above critical torque (Pethick et al., 2016). Although the fatigue protocol contractions were performed above critical torque (60% of MVC), the comparison of fresh and fatigued torque complexity was derived from contractions below KE critical torque (20% of MVC). The lower intensity was selected to maximize the number of MUs decomposed from the contraction. However, it is conceivable that fatigue‐related changes in torque complexity were influenced by the contraction intensity. Further research is required to elucidate the relationship between torque complexity and common synaptic input across a range of isometric contraction intensities.

In this study, we did not measure the development of central and peripheral fatigue induced by the fatigue protocol. Given that the contraction intensity certainly exceeded critical torque, we can reasonably infer that both central and peripheral fatigue did occur (Pethick et al., 2016). However, the absence of a quantitative measure of fatigue limits our ability to explain in full the impact of inter‐individual levels of fatigue on torque complexity and the strength of common synaptic input.

## 5 CONCLUSION

The ability to modulate common synaptic input to the motoneurons is an adaptive neural strategy required for continued torque production when the muscle is fatigued. The present study presents the first evidence that demonstrates an association between the strength of common synaptic input in the alpha (physiological tremor) band and the complexity (CI‐28 and DFA) of isometric KE torque signals derived from fresh and fatigued muscle contractions. Furthermore, the study presents new evidence of a neuromuscular fatigue‐related cross‐over in the MSE curves derived from isometric KE torque. Importantly, from the point of cross‐over in the MSE curves (scales 11–28), the fatigue‐related increase in SampEn was significantly predicted by the fatigue‐related increase in the strength of alpha band oscillations in common synaptic input. This suggests that higher‐frequency oscillations in common synaptic input might be embedded in the low‐frequency components of common neural drive and captured as the greater irregularity of the low‐frequency components of the fatigued torque signals (i.e., the higher SampEn at longer scales of the MSE curves).

This study provides experimental data showing that the complexity measures (MSE, DFA and CI‐28) derived from isometric KE torque signals reflect the neural mechanisms underpinning the modulation of torque control and therefore the strategy of the neuromuscular system to adapt muscle torque to meet task demands (Manor & Lipsitz, 2013; Peng et al., 2009). These findings also support the theoretical framework of physiological complexity, which proposes that the changes in a system at a component level (system inputs) are reflected at a behavioural level in the complexity of the output of the system (Lipsitz, 2002; Sleimen‐Malkoun et al., 2014; Vaillancourt & Newell, 2002).

## AUTHOR CONTRIBUTIONS

Christopher R. J. Fennell, James G. Hopker and Alexis R. Mauger designed the research. Christopher R. J. Fennell conducted the experiments, data collection and data analysis. Christopher R. J. Fennell, James G. Hopker and Alexis R. Mauger wrote the manuscript. All authors approved the final version of the manuscript and agree to be accountable for all aspects of the work in ensuring that questions related to the accuracy or integrity of any part of the work are appropriately investigated and resolved. All persons designated as authors qualify for authorship, and all those who qualify for authorship are listed.

## CONFLICT OF INTEREST

The authors report no conflicts of interest or competing interests.

## Supporting information

Supplementary Material

## Data Availability

Data are available upon reasonable request.
